# Potential Risks to Human Health Caused by the Use of Pesticides in Soils of Three Municipalities Impacted by Localized Malaria in the Brazilian Amazon

**DOI:** 10.3390/toxics13100900

**Published:** 2025-10-21

**Authors:** Letícia Furtado dos Santos, Ricardo Jorge Amorim de Deus, Izis Mônica Carvalho Sucupira, Davi do Socorro Barros Brasil, Rosivaldo de Alcântara Mendes

**Affiliations:** 1 Institute of Exact and Natural Sciences, Federal University of Pará, Belém 66075110, Brazil; 2 Environmental Section, Evandro Chagas Institute, Ananindeua 67030000, Brazil; rosivaldomendes@iec.gov.br; 3 Faculty of Biotechnology, Federal University of Pará, Belém 66075110, Brazil; dedeus@ufpa.br; 4 Parasitology Section, Evandro Chagas Institute, Ananindeua 67010030, Brazil; izissucupira@iec.gov.br; 5 Institute of Technology, Federal University of Pará, Belém 66075110, Brazil; davibb@ufpa.br

**Keywords:** DDT, alphacypermethrin, soil, malaria, health risks, ecological risk, monitoring

## Abstract

Dichlorodiphenyltrichloroethane (DDT), used in the 20th century to combat malaria, is considered harmful to health and the environment. As an alternative, insecticides such as pyrethroids have been used, especially alphacypermethrin, which is applied in mosquito nets impregnated with long-lasting insecticide (LLIN). This study analyzed the concentrations of DDT and alphacypermethrin in soils from three municipalities in the Legal Amazon (Mazagão, Porto Velho, and Cantá) using gas chromatography. The results showed the presence of DDT and metabolites, indicating slow degradation in the region, especially in Cantá, with an average of 2.694 mg/kg of total DDT. Alphacypermethrin stands out in Porto Velho, with an average of 0.364 mg/kg, possibly due to the use of LLINs. DDT did not represent a significant ecological risk in this study, but it did present risks to human health, mainly through food intake. The incremental lifetime cancer risk (ILCR) indicated potential danger, with values of up to 2.93 × 10^−3^ for DDT and 1.17 × 10^−1^ for alphacypermethrin. The total non-carcinogenic risk index (HI) was extreme, with a maximum value of 336.61. The pesticides evaluated did not present an ecological risk, but they do pose risks to human health, indicating irregular use of LLINs and the need for continuous monitoring.

## 1. Introduction

Dichlorodiphenyltrichloroethane (DDT) is a pesticide that belongs to the organochlorine group. It has high chemical stability, low solubility in water, and high solubility in animal fat [1,2,3]. In the air, DDT decomposes rapidly in sunlight, while in the soil, its decomposition occurs slowly, which means it remains in the environment for a long time. Its main degradation products are dichlorodiphenyldichloroethane (DDD) and dichlorodiphenyldichloroethylene (DDE) [4,5].

DDT was one of the most widely used and studied synthetic substances of the 20th century. Its high efficiency in eliminating insects made it an important ally in the fight against diseases such as malaria, considered one of the persistent endemic health problems in Brazil and worldwide. Its use during campaigns carried out from the 1940s to the 1960s led to a sharp drop in cases of this disease, which was even eradicated in certain regions [6,7].

It is now known that the use of this insecticide was not a sustainable practice. DDT is harmful to health and the environment, classified as a Persistent Organic Pollutant (POP), and has the potential to remain in the environment for long periods due to its low vapor pressure and resistance to photo-oxidation, to accumulate in the food chain, and to be transported to locations far from its release through the air, water, and even species that migrate from their habitat. This poses risks to both human health and the environment. Even after its ban, DDT may still be present, especially in soil [8,9,10,11,12]. The main form of human exposure is through the ingestion of contaminated food, especially fatty foods of animal origin and breast milk, which can cause cancer, neurological damage, and reproductive disorders [10,13]. Given this scenario, other groups of pesticides have been implemented in malaria campaigns, which are supposed to pose fewer risks to human health and the environment, notably pyrethroids [4,14,15].

Pyrethroids are synthetic derivatives of pyrethrins, toxic esters isolated from the flowers of *Chrysanthemum cinerariaefolium* species. They have low environmental impact and toxicity in mammals and are effective against a wide range of insects [16,17,18]. Among this group of insecticides, alphacypermethrin (C_22_H_19_Cl_2_NO_3_) is one of the most notable pyrethroids, as it is the most widely used for domestic use and recommended by the WHO, effective in combating a wide range of insects. It is classified as a type II pyrethroid, which gives it greater insecticidal potency [19,20].

It is used in communities in various ways, including indoor spraying, fogging, and impregnation of mosquito nets, a method better known as long-lasting insecticide-treated mosquito nets (LLINs). Its residual action can last for up to 20 washes, with an estimated useful life of 5 years. These mosquito nets are made of synthetic polyethylene fabric, impregnated with concentrations of around 6700 mg/kg of alphacypermethrin. LLINs represent a physical and chemical barrier between people and malaria vectors, and their use has contributed to the decline in malaria cases in Brazil [21,22].

This pyrethroid does not cause harm to human health, as mammals have a faster metabolic elimination rate, are fat-soluble, and are less susceptible to the toxicity of this contaminant. However, it is harmful to fish, bees, and aquatic arthropods and can cause damage such as imbalance in the food chain, as well as accidentally exposing other species [16,23].

Malaria is considered a persistent public health problem worldwide. In Brazil, it mainly affects the Amazon region, which accounts for 99.98% of cases due to its hot and humid tropical climate, with the highest incidence rates in the states of Acre, Amazonas, Pará, Amapá, Rondônia, Roraima, Tocantins, Mato Grosso, and Maranhão [24]. According to the latest epidemiological bulletin from the Secretariat of Health and Environmental Surveillance, updated in 2024, mining areas were the only ones where autochthonous cases of malaria increased from 2021 to 2022, particularly in the states of Roraima (91.5%), Amapá (28%), and Rondônia (6.4%) [25].

This study aims to determine the levels of two pesticides, DDT and alphacypermethrin, in the soil of three areas of the Brazilian Amazon region that are affected by malaria, as well as to assess the risks to human health associated with exposure to these contaminants.

## 2. Materials and Methods

### 2.1. Study Area

**Figure 1:**
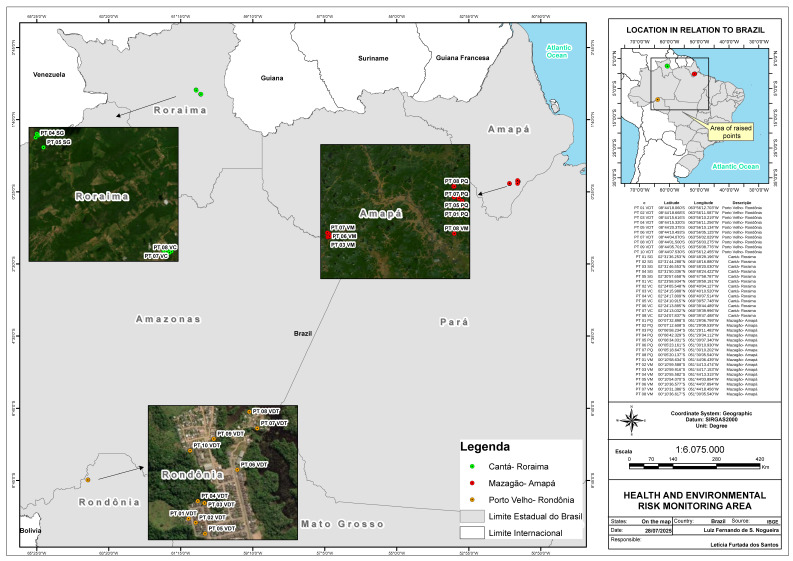
Location of sampling sites in the communities of Piquiazal (PQ) and Vila Maracá (VM) in Mazagão, Vila Dnit community (VDT) in Porto Velho, and Vila Central (VC) and Serra Grande (SG) in Cantá. QGIS software version 3.38.0 Grenoble was used to construct the map.

Samples were collected in three municipalities in the Brazilian Amazon: in Mazagão, in the state of Amapá, in the communities of Piquiazal (PQ) and Vila Maracá (VM); in Porto Velho, in the state of Rondônia, in the community of Vila Dnit (VDT); and in Cantá, in the state of Roraima, in the localities of Vila Central (VC) and Serra Grande (SG) (Figure 1). All municipalities used DDT in past decades, received LLINs distributed by the Ministry of Health and/or State Health Secretariats, and had an annual parasite incidence (API) that classified them as medium (10 to 49.9) or high risk (≥50) [25].

Mazagão is a municipality in the state of Amapá, with a land area of 13,294.778 km^2^, located approximately 35 km from the city of Macapá, the state capital. It has an altitude of 7 m, which is typical of many areas in the Amazon region, with the following geographical coordinates: Latitude: 0°6′58″ South, Longitude: 51°17′10″ West, located very close to the Equator. According to the latest IBGE census conducted in 2022, there were an estimated 23,575 inhabitants in 2024, a population density of 1.65 inhabitants/km^2^, which indicates a relatively dispersed population in its area, and an IDHM of 0.592. The climate of this region is tropical, with October being the driest month and April the rainiest, with precipitation of 25 mm and 349 mm, respectively [26,27].

According to the latest update of the list of municipalities belonging to areas at risk or endemic for malaria, between 2021 and 2023, the average number of malaria cases in Mazagão was 143 [28]. Socio-environmental characteristics contribute to the proliferation of this disease, especially when it involves migration flows between urban and rural areas. Rural areas, in particular, contribute most to the number of reported cases, as they are mostly made up of riverside areas surrounded by forest and floodplains, which are ideal environments for the malaria vector [29].

Porto Velho is the capital of the state of Rondônia, located on the right bank of the Madeira River, characterized as the largest tributary of the right bank of the Amazon River. The city is 87 m above sea level, with the following geographical coordinates: Latitude: 8°45′43″ South, Longitude: 63°54′14″ West. The municipality has a territorial area of 34,091.146 km^2^, in addition to bordering Bolivia. The latest IBGE census estimated 514,873 inhabitants, with a population density of 13.51 inhabitants/km^2^ and an IDHM of 0.736 [30,31].

The rainy season in the region begins in October and continues until April of the following year. June to August is considered the dry season, and May and September are transition months. Climate has a strong influence on malaria, which affected an average of 7449.70 people (referring to the years 2021, 2022, and 2023). The rainy season directly induces the formation of breeding sites, an environment conducive to the proliferation of the Anopheles mosquito, which transmits the disease [24,31,32].

Cantá is a municipality located in the central–eastern part of the state of Roraima, a Brazilian state situated between Venezuela and Guyana, and is notable for its rich diversity of vegetation and water resources. Its territorial area is equivalent to 7664.831 km^2^, with an estimated 20,552 inhabitants, a population density of 2.44 inhabitants/km^2^, and an IDHM of 0.619. The region’s climate is humid tropical, with a dry season between January and March and a rainy season from April to August. This locality has a significant level of malaria infection, according to the latest epidemiological bulletin, which shows an average of 840.3 cases during the years 2021 to 2023 [24,33,34,35].

### 2.2. Sample Collection and Analysis

Sampling was conducted in 2023, with a total of 39 samples collected: 16 in the municipality of Mazagão, 10 in Porto Velho, and 13 in Cantá. Collections were conducted exclusively in the homes of individuals who received and used mosquitoes impregnated with long-lasting insecticides to combat malaria, which limited the number of households eligible for participation in the evaluation.

Soil samples were collected from a 10 cm surface layer, totaling 1 kg per sample, using a stainless-steel hand-held Dutch auger. The samples were placed in chemically cleaned polyethylene bags and transported to the laboratory, where they were immediately stored under controlled conditions. In the laboratory, the samples underwent a preliminary preparation procedure that included sectioning, disaggregation, sieving, and separation of the coarse fraction and particles up to 270 mesh.

The following DDT metabolites were evaluated: *p,p′*-DDT, *o,p′*-DDT, *p,p′*-DDE, *o,p′*-DDE, *p,p′*-DDD, and *o,p′*-DDD. These compounds are persistently detected in environmental matrices due to their chemical stability, lipophilicity, and long half-life, characteristics that favor their bioaccumulation and biomagnification in food chains. In addition, these metabolites are often used as environmental markers of DDT exposure, since their presence indicates both the historical use and persistence of the original compound in the environment.

For the extraction process of these analytes, the samples were subjected to a microwave-assisted extraction (MAE) system, in which 1 g of sample was used for extraction, with the addition of 15 mL of n-hexane/acetone (1:1; *v*/*v*). The samples are heated to an initial temperature of 30 °C for 2 min, then gradually heated to 100 °C (10 °C min^−1^) and left for 20 min at a power of 800 W. After microwave extraction, the samples undergo a clean-up process using cartridges packed with 2 g of silica gel and 1 g of anhydrous sodium sulfate. The cartridges are homogenized with 2 mL of hexane; then, the extracted samples are inserted, followed by 10 mL of hexane/acetone (1:1; *v*/*v*), 10 mL of dichloromethane/hexane (1:9; *v*/*v*), and 10 mL of hexane. After this procedure, the samples are concentrated in a vacuum evaporator for 1 mL.

The insecticides were determined using gas chromatography, using a Trace GC Ultra gas chromatograph (Thermo Scientific, Waltham, MA, USA) equipped with an electron capture detector (GC-ECD) under the following conditions: 30 m long capillary column, 0.25 mm internal diameter, and 0.25 µm film thickness, with the column oven temperature ramp at 200 °C (12 °C/min) for 2 min and 280 °C (10 °C/min) for 8 min. The carrier gas was nitrogen (N_2_) (99.999% purity) with a flow rate of 1.5 mL/min. The injector was operated at 250 °C in splitless mode, and the detector temperature was 300 °C with an injection volume of 1 µL. The results were confirmed using gas chromatography coupled with triple quadrupole mass spectrometry (GC-MS/MS) with the following chromatographic conditions: 30 m long capillary column with an internal diameter of 0.25 mm and a film thickness of 0.50 μm. The oven ramp temperature for the column was maintained at 80 °C for 1 min and 80 °C to 280 °C (13 °C/min) for 3.5 min. The carrier gas was ultra-pure helium with a flow rate of 1 mL/min. An injector was used with a temperature of 280 °C in the splitless mode. The transfer line temperature was 275 °C, and the ion source temperature was 260 °C.

Blank analyses were performed to evaluate possible interferences in the sample matrix, in addition to triplicate recovery tests using fortified samples and standard reference material (SRM 1944, NIST, Gaithersburg, MD, USA). Soil samples were spiked with 50 µg/g of the pesticide suite, including *op*- and *pp*-DDT, DDE, DDD, and alphacypermethrin. The recovery results obtained for the fortified samples and reference materials ranged from 83.2% to 94.6% and from 73.5% to 88.7%, respectively, remaining within the acceptable range for chromatographic analysis (70% to 120%). The detection limit (LD) of the method was determined by the ratio between the signal and noise in the baseline of the chromatogram, considering the lowest concentration of the fortified standard detectable by the equipment, ranging from 0.0001 to 0.005 mg/kg. The limit of quantification (LQ) was calculated as 3.3 times the LD value, according to the recommendations of DOQ-CGCRE-008 [36], resulting in concentrations ranging from 0.0003 to 0.0165 mg/kg.

### 2.3. Determination of pH

Ten grams of soil were weighed into a 100 mL beaker, 25 mL of deionized water was added, and the sample was stirred with a glass rod and left to stand for one hour. After that, each sample was stirred again with a glass rod, and the pH meter electrodes were immersed in the homogenized suspension for subsequent pH reading [37].

### 2.4. Organic Matter Analysis

To determine the organic matter content, the combustion method was used with a muffle furnace. To do this, it was necessary to weigh approximately 5 g of the soil sample in a porcelain capsule. Next, it was subjected to a temperature of 105 °C for 2 h in an oven. The capsule was then removed and placed in a desiccator to cool for approximately 15 min, after which it was weighed. Subsequently, it was subjected to a temperature of 550 °C for 3 h in a muffle furnace, followed by conditioning in the desiccator until the sample cooled, and then weighed again. The percentage of organic matter (OM) content was calculated according to Equation (S1), shown in the Supplementary Materials [37,38].

### 2.5. Granulometric Analysis

To determine the granulometry of the soil, the samples were dried at room temperature, after which the material was deposited on a 2 mm mesh, and the aggregates were broken manually. The set of sieves with mesh openings of 1.8, 0.5, 0.25, 0.15, 0.125, 0.075, 0.053, 0.045, and 0.038 mm, clean and dry, was weighed, as well as approximately 20 g of the sample, and added to the set assembled in descending order. The set was shaken for 5 min using a sieve shaker. After this period, the set of sieves was weighed again (sieve + sample), and the granulometric curve was plotted [39,40].

### 2.6. Ecological Risk Analysis

The ecological risk assessment was conducted according to the Canadian Soil Quality Guidelines for the Protection of Environmental and Human Health [41]. This methodology establishes soil quality parameters based on guideline values for contaminant concentrations, aiming to protect terrestrial ecosystems and prevent adverse impacts on human health.

The analysis was based on a normative table in the guidelines for total DDT, which provides maximum acceptable limits for chemicals in soil (Supplementary Materials Table S1). These reference values served as a comparative criterion for interpreting the data obtained from the samples and allowed us to estimate the degree of potential ecological risk associated with the areas assessed. There are no reference values for the pesticide alphacypermethrin.

### 2.7. Human Health Risk Assessment

A probabilistic model was developed to assess health risks using the Monte Carlo simulation technique. The objective was to measure the risk of cancer and other non-carcinogenic risks related to human exposure to DDTs and alphacypermethrin, which can be transmitted through the soil. To this end, a sensitivity analysis was performed, which helped us to understand the impact of uncertainties in the input data and identify which factors most influence the risk. Following the guidelines of the Exposure Factors Handbook [42], the average daily dose (ADD, mg/kg/day) of DDTs and alfa-cypermethrin transmitted through the soil was calculated, considering two routes of exposure: soil ingestion (ADDInt_soil) and food ingestion (ADDInt_food). The equations used are presented in Equations (S2) and (S3) of the Supplementary Materials, as well as the results, represented in Figure S1.

In addition, the risk was assessed based on the Guide for Conducting Risk-Based Corrective Actions (RBCAs), in accordance with the protection of human health and the environment. The daily intake of DDT and alphacypermethrin through food was calculated based on the parameters established by the American Society for Testing and Materials (ASTM) tests, according to the Standard Guide for Risk-Based Corrective Action [43]. Using data from the Intergovernmental Forum on Chemical Safety [44], values were estimated for the mathematical model of daily intake, considering foods such as tomatoes, onions, cabbage, peppers, cucumbers, and lettuce, among others. The bioconcentration values for these foods are available in Table S2 of the Supplementary Materials [45].

To assess cancer risk, indices such as lifetime average daily dose (LADD) and lifetime incremental cancer Risk (ILCR) were used. These indices follow the standards of the US Environmental Protection Agency (USEPA), with LADD and ILCR values < 1 × 10^−4^ (acceptable, no cancer risk) and LADD and ILCR > 1 × 10^−4^ (unacceptable, cancer risk) [46], as shown in Equation (S4) of the Supplementary Materials.

The non-carcinogenic risk was assessed by calculating the hazard quotient (HQ) using the exposure routes of soil ingestion (HQInt_soil) and food ingestion (HQInt_food), as shown in Equation (S5). To estimate the total non-carcinogenic risk, the hazard index (HI) was used, as shown in Equation (S6).

Finally, health risks were categorized and estimated for three age groups: children (1 to 11 years), adolescents (12 to 17 years), and adults (18 to 70 years).

### 2.8. Statistical Analysis

For the statistical treatment of the results, the measures of central tendency (mean and median), dispersion (standard deviation), and Pearson’s correlation were calculated. For multivariate analysis, principal component analysis (PCA) was applied. The statistical tests were performed using Minitab version 14 [47,48].

## 3. Results and Discussion

### 3.1. Evaluation of DDT and Its Metabolites

The descriptive statistics for the parameters analyzed are presented in Table 1. The highest means and maximum values of the analytes evaluated were identified in the municipality of Cantá, in the state of Roraima. On the other hand, the minimum values observed at all study sites were below the detection limit of the method (<LD). The detailed results are described in the Supplementary Materials Table S3.

**Figure 2:**
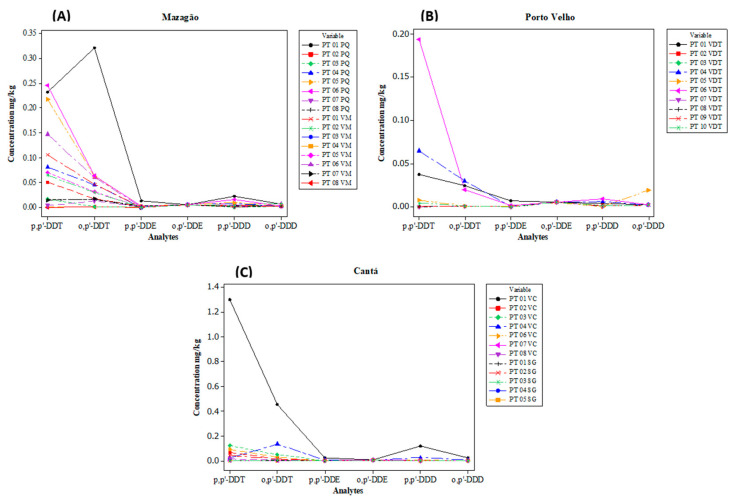
Concentration of DDT and its metabolites in mg/kg in the communities of Piquiazal (PQ) and Vila Maracá (VM) in Mazagão (**A**), Vila Dnit community (VDT) in Porto Velho (**B**), and Vila Central (VC) and Serra Grande (SG) in Cantá (**C**).

In Mazagão (Figure 2A), concentrations of *p,p′*-DDT, *o,p′*-DDT, *p,p′*-DDD, and *p,p′*-DDE were found. The highest level was found in *o,p′*-DDT (0.320 mg/kg) at location PT 01 PQ. At this same site, significant levels of *p,p′*-DDT (0.231 mg/kg), *p,p′*-DDD (0.022 mg/kg), and *p,p′*-DDE (0.013 mg/kg) were also found.

In Porto Velho (Figure 2B), sampling site PT 06 VDT, located in the Vila Dnit community, presented the highest concentrations with 0.193 mg/kg, 0.019 mg/kg, 0.001 mg/kg, and 0.009 mg/kg for *p,p′*-DDT, *op′*-DDT, *p,p′*-DDE, and *p,p′*-DDD, respectively.

In Cantá (Figure 2C), the highest concentrations of DDTs in this study were found, particularly at sampling site PT01 VC, located in the Vila Central community, which had concentrations of 1.297 mg/kg (*p,p′*-DDT), 0.452 mg/kg (*o,p′*-DDT), 0.024 mg/kg (*p,p′*-DDE), 0.119 mg/kg (*p,p′*-DDD), and 0.023 mg/kg (*o,p′*-DDD). Despite the considerable values of these analytes, all maximum values are within the limits permitted by CONAMA, for residential soils (Table 1) [49].

Despite the ban on DDT decades ago, the results of this study show that it is still present in the soil. A soil is the main repository of this pesticide, since its half-life, which is equivalent to 1025 days, is higher than that of air (t½ = 1.4 days) and sediment (t½ = 171 days), making soil contamination by this pesticide a potential source of contamination for air and water [50].

Investigations in communities in Chiapas, Mexico, detected total DDT concentrations in soil of up to 26.980 mg/kg, higher than those observed in this study [51]. In contrast, assessments carried out in communities in Tabasco, Mexico, identified maximum values of 0.123 mg/kg of total DDT, lower than those found in this study, both related to the use of the insecticide to combat malaria [52]. On the other hand, assessments in urban soil in the city of Kurukshetra, Haryana, India, revealed significant concentrations of total DDT at 37.420 mg/kg [53]. In addition, in the city of Belém, Pará, Brazil, obsolete DDT storage deposits showed high concentrations of up to 1887.00 mg/kg, evidencing high persistence of the contaminant in deactivated storage environments [54].

The results identified for DDT and its metabolites in this study are below those detected in the city of Kurukshetra, Haryana, India [50], where concentrations were found of up to 10.9 mg/kg, 7.06 mg/kg, 18.03 mg/kg, and 8.49 mg/kg for *o,p′*-DDT, *p,p′*-DDT, *p,p′*-DDD, and *p,p′*-DDE, respectively. However, it should be noted that environmental characteristics such as temperature, humidity, and organic carbon content contribute to the degradation process, so that each study region will have its own specific decomposition period [50].

At PT 01 VC in Cantá, the high DDT concentrations may be related to the soil’s physicochemical characteristics, such as its acidic pH, which had a mean value of 5.22 ± 1.11, with 87% of the samples having a pH below 6. This acidic pH condition can affect the behavior of pesticides in the soil, as it favors adsorption processes due to the higher concentration of the pesticide by positively or negatively charged particles, depending on their acid-base conjugation state. This factor can increase the retention of the contaminant in the soil matrix, environmentally facilitating its mobilization to surface waters through leaching processes [55].

Furthermore, the low organic matter (OM) trapped in the soil, approximately 3.31%, can negatively influence pesticide handling processes, as OM plays a fundamental role in microbial activity and contaminant classification. An acidic soil pH can also affect microbial activity, as acidic conditions can reduce the population of microorganisms responsible for biodegradation [56]. Lower OM levels also reduce the variety of pesticides, increasing their transport availability and, consequently, their persistence in the environment. Because DDT is highly lipophilic and poorly water soluble, its handling in soil is limited, favoring its persistence and leading to pollution of other environmental compartments, such as the atmosphere through soil–air gas exchange and water bodies through surface runoff [5,57,58].

A soil particle size analysis revealed that the region predominantly contains sandy particles with an average diameter of approximately 1.18 mm, a common characteristic in the Amazon region. The complete results of the particle size analyses are presented in the Supplementary Materials (Figures S2–S4) [40]. Sandy soils tend to be acidic and have low natural fertility due to nutrient scarcity, although they may be relatively fertile due to the accumulation of organic matter from leaves, fruits, and animal remains, forming a humus layer. However, their high permeability favors the percolation of water and contaminants, increasing the risk of pesticide leaching into water bodies [59,60].

Therefore, soil physicochemical conditions, including an acidic pH, low OM, and sandy texture, influence the transport, retention, and persistence of pesticides, contributing to the high DDT concentrations observed and increasing the risk of environmental contamination. It should be noted that, for a more in-depth understanding of pesticide behavior in this environment, additional studies of sorption/desorption, microbial manipulation, and transport dynamics would be necessary, as these aspects were not included in this study.

### 3.2. Analysis of Correlations Between DDT, pH, and OM

In order to identify possible relationships between the DDT results and the soil pH and organic matter (OM) data, Pearson’s correlation was performed, adopting a significance level of *p* ≤ 0.05 (Table 2). The correlation was not applied to the op′ DDE compound, since it did not present detectable concentrations in any of the sampled sites.

The results presented in Table 2 show a very strong and positive correlation of the analyte *p,p′*-DDD with *p,p′*-DDT and *p,p′*-DDE, as well as of *p,p′*-DDE with *o,p′* -DDT. On the other hand, *p,p′*-DDT showed a strong correlation with *o,p′*-DDT and *p,p′*-DDE, as did op′ DDT. Despite the affinity that DDT generally has with organic matter, due to the fact that it is a hydrophobic organic compound, it did not show a correlation with this parameter and with pH [11]. However, these parameters showed a moderate correlation with each other, which can be observed in the principal component analysis (PCA) (Figure S5, Supplementary Materials).

Contaminants that showed a very strong correlation formed a cluster. However, *o,p′*-DDD was further away from the other analytes in PCA, as it did not show concentration for most sites, so its correlation was moderate with DDT and its other isomers.

### 3.3. Degradation Conditions and Potential Sources of DDT

**Figure 3:**
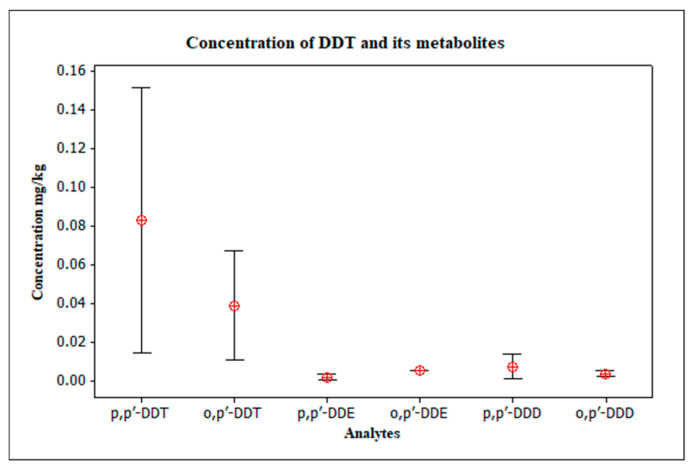
Concentration of DDT and its metabolites detected.

In the overall assessment of DDT and its metabolites in the study areas, DDT was predominant in relation to its main degradation products (Figure 3), something also found in the investigation of soils near the Tapajós and Madeira rivers in the Amazon region [61], as well as in assessments carried out on residential soils in the Lake Puruzinho region, in Amazonas [62].

Given that DDT, depending on how long it has been applied to the environment, slowly degrades into DDD and DDE, this high proportion of DDT through its degradation products may be indicative of recent contamination by this pollutant. To evaluate this suspicion, the following ratio was calculated: (*p,p′*-DDE + *p,p′*-DDD)/*p,p′*-DDT; if the value obtained is >1, it indicates long-term contamination, but if the ratio is <1, it indicates recent contamination at the site [63].

**Figure 4:**
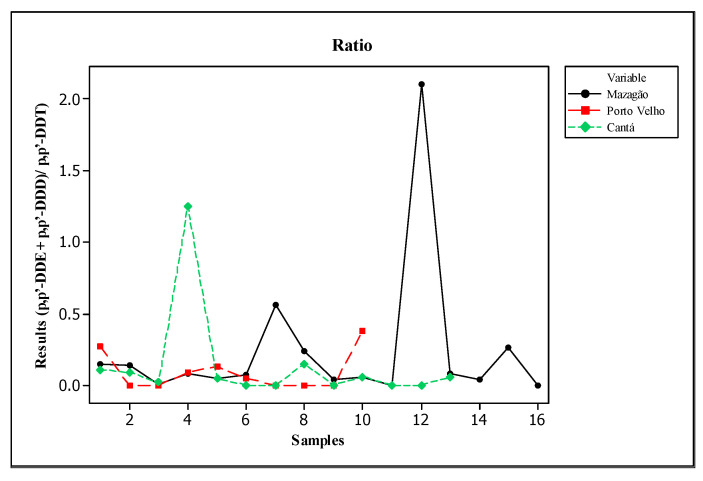
Values found using the ratio (*p,p′*-DDE + *p,p′*-DDD)/*p,p′*-DDT.

In all areas analyzed, except sites PT04 VM in Mazagão and PT04 VC in Cantá, the ratio (*p,p′*-DDE + *p,p′*-DDD)/*p,p′*-DDT) presented values lower than 1, as shown in Figure 4. Considering that commercial DDT is mainly composed of *p,p′*-DDT (77.1%), followed by *o,p′*-DDT (14.9%), *p,p′*-DDE (4%), *p,p′*-DDD (0.3%), *o,p′*-DDD (0.1%), *o,p′*-DDE (0.1%), and impurities (3.5%) [53], and the fact that it was banned in Brazil for any epidemiological use in 2009 due to its adverse effects on the environment and human health, the results indicate a slow degradation of this pesticide in the region, considering its intensive use in the past to combat malaria [50,64,65]. In addition, the proximity of the study sites to former DDT storage depots containing obsolete stocks may have contributed to the persistence of this pesticide, as many of them were abandoned and left with considerable amounts of this insecticide [66,67].

As this is the first study on DDT levels in areas with the highest incidence of malaria in the Amazon over the years, there are no previous data available on the concentrations of this compound in these locations. In this context, the data obtained contribute significantly to the understanding of the persistence of DDT in the Amazonian environment and to the formulation of strategies for monitoring and mitigating the associated risks.

The degradation of DDT into DDE or DDD depends significantly on aerobic and anaerobic conditions. DDT is dechlorinated to DDE under aerobic conditions and dechlorinated in the reductive process to DDD under anaerobic conditions. To identify the main contribution of these conditions, the DDE/DDD ratio was calculated. When the result of this ratio is less than 1, it points to the reductive dechlorination of the original DDT, which can occur through microbial or chemical pathways [63].

**Figure 5:**
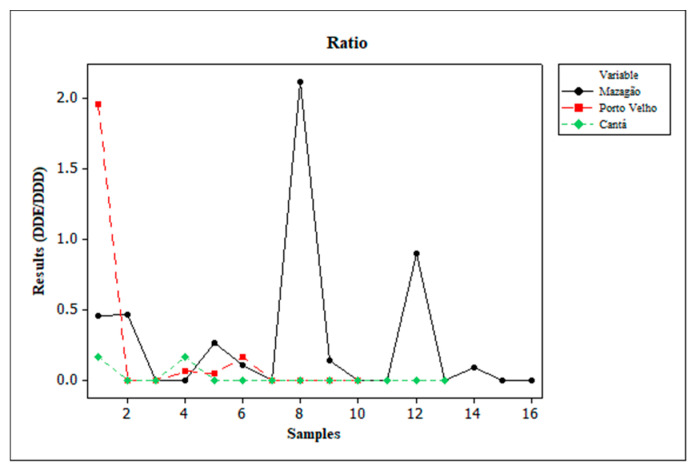
Values found using the DDE/DDD ratio.

Except for sites PT 08 PQ in Mazagão and PT 01 VDT in Porto Velho, all regions studied had DDE/DDD ratios below 1 (Figure 5), results similar to those found in studies conducted in Kurukshetra, Haryana, India [53]. This indicates that there is a higher concentration of DDD compared to DDE in the soil. This observation suggests that, in the sites analyzed, anaerobic conditions play a significant role in the reductive dechlorination of DDT. Anaerobic degradation is possibly favored by flooding caused by rainfall. This relationship is associated with the fact that sample collections occurred during the rainy season in the study regions [50,63,66].

### 3.4. Evaluation of Alphacypermethrin

**Figure 6:**
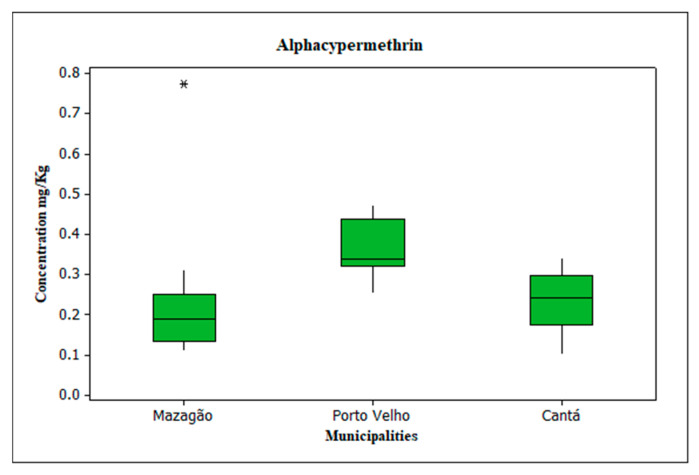
Concentration of alphacypermethrin in mg/kg. Legend: * Outlier referring to the maximum value presented at site PT 03 VM.

Significant concentrations of alphacypermethrin were identified in the soil (Figure 6), with the highest levels identified in the community of Vila DNIT in Porto Velho, with an average of 0.364 mg/kg, a maximum of 0.473 mg/kg, and a minimum of 0.252 mg/kg, followed by the municipality of Cantá with an average of 0.230 mg/kg, a maximum of 0.341 mg/kg, and a minimum of 0.100 mg/kg, while the region of Mazagão had an average of 0.226 mg/kg and an outlier due to the maximum value presented at site PT 03 VM, which was 0.775 mg/kg, while the minimum value for this municipality was 0.109 mg/kg. Pearson’s correlation with a significance level of ≤0.05 showed an inversely proportional correlation with pH and OM, in which the former was moderate (*p* = −0.439) and the latter was weak (*p* = −0.382). Thus, it can be seen that these parameters have little influence on the action of alphacypermethrin in the study area. Furthermore, no statistically significant correlation was observed between DDT and its metabolite levels and alphacypermethrin, as *p*-values were above the established significance threshold. Therefore, the distribution of alphacypermethrin levels in the soil was independent of DDT levels. The highest alphacypermethrin levels were identified in the municipalities of Porto Velho, Cantá, and Mazagão, in decreasing order, while for DDTs, the distribution of levels followed the sequence of Cantá, Mazagão, and Porto Velho, also in decreasing order.

The concentrations measured in the municipalities indicate recent application of this insecticide, given that it has a half-life ranging from 3 to 96 days under aerobic conditions and from 5 to 430 days under anaerobic conditions [68].

These concentrations may originate mainly from mosquito nets impregnated with alphacypermethrin, used by the population of the communities evaluated and also by public health campaigns using this pesticide. It was observed that some residents are unaware of the correct way to handle and use impregnated mosquito nets. On the other hand, even among those who demonstrate knowledge of the proper guidelines, there are cases in which the instructions are not followed, resulting in inappropriate and negligent practices. Among these practices, the use of mosquito nets to cover crops, fishing activities, improper storage, and excessive washing in inappropriate places, such as rivers and lakes near homes, stand out. In some cases, mosquito nets are washed in the same environments used for washing dishes or together with other family clothes. Such behaviors can compromise the health of residents and the quality of the local soil, which is often used for subsistence activities.

The degradation of alphacypermethrin in soil can be influenced both by the chemical action of contaminants in this matrix and by the presence of living organisms. Studies on the influence of trace metals, Cu^2+^, Zn^2+^, Cd^2+^, and Fe^2+^, on the photodegradation of alphacypermethrin in agricultural soil have revealed that the degradation/persistence of this insecticide was affected in the same proportion as the increase in metal concentrations in this matrix [69]. Other studies have found, through experiments on the action of cypermethrin on earthworms, that they can be an important ally in the degradation process, as they have the ability to absorb and accumulate residues of this pesticide in the soil. Their action catalyzes the degradation process of cypermethrin, in which a variation in half-life of only 26.6 to 27.7 days was identified. However, earthworms, like microorganisms that live in the soil, are negatively affected by the effects of this pesticide, which can harm the food chain and biodiversity [70].

Studies show that soil is the main reservoir of pesticide residues, which can result in toxicity for terrestrial organisms and can spread to water bodies, reaching rivers, lakes, and streams through surface runoff. Occasionally, this becomes a risk to aquatic organisms, especially fish, since these individuals do not have the carboxylesterase enzymes for the hydrolysis of pyrethroids, which means that the biotransformation and detoxification processes of this insecticide occur slowly compared to other aquatic organisms [71,72].

Soil contaminated with alphacypermethrin also poses a potential risk to flora, as plant roots can absorb this contamination and migrate to consumable parts, exposing the population to contaminated food, especially that consumed raw, as observed in research conducted on farms in Santa Maria, Pangasinan, in the Philippines, where the presence of several insecticides, including the pyrethroid cypermethrin, was detected in eggplant [71].

Considering that few studies have been conducted to evaluate alphacypermethrin in soil, its quantification in this matrix is essential to verify the possible ecotoxicological impacts of this insecticide on the environment, due to its widespread and intense use today, which poses a potential threat to soil organisms, as they use this matrix for habitat and food, as well as other environmental matrices that are part of the environment.

### 3.5. Results of Ecological Risk Analysis

When assessing ecological risks, it was observed that only the municipality of Cantá presented a site with potential ecological risk for the region, as the SQGE value was higher than 0.7 mg/kg. The other sites were considered ecologically safe, since their SQGE values are within the acceptable range, i.e., below 0.7 mg/kg, according to the parameters established by CCME [41]. The total DDT values that were used to perform this evaluation can be seen in Table S3 of the Supplementary Materials.

Soil contamination, especially at this site in Cantá, can pose risks to animals that consume food in direct contact with this matrix, as well as to plants present in this environment. This is because both soil and living organisms, such as animals and plants, can act as potential matrices of ecological risk caused by the insecticide [73].

It is worth noting that DDT poses a considerable ecological risk due to its toxic effects on the ecosystem, as it can be transmitted from one organism to another through the food chain. This is because DDT has the potential for biomagnification, meaning it can accumulate in the adipose tissue of living organisms, increasing its impact throughout the chain [74].

### 3.6. Human Health Risk Analysis

#### 3.6.1. Variations in Carcinogenic and Non-Carcinogenic Health Risks

The analysis of the analyte *p,p′*-DDT showed the highest ILCR indices for both soil and food intake. The highest estimate of carcinogenic risk from soil ingestion was observed in adolescents in the community of Vila Central, municipality of Cantá, with a value of 4.79 × 10^−8^, followed by Mazagão (Piquiazal) with 2.68 × 10^−8^, and Porto Velho (Vila Dnit) with 7.69 × 10^−9^ (Table S4). All these values remain within the established acceptability limit (ILCR < 1 × 10^−4^), indicating an acceptable carcinogenic risk. These results are consistent with studies conducted in China, where DDT values reached an index of 2.21 × 10^−9^, suggesting that exposure to DDT through soil ingestion in the study area is unlikely to pose a significant long-term health threat to the groups evaluated [12].

On the other hand, in the food intake route, the ILCR reached higher values in children, reaching 2.93 × 10^−3^ in Vila Central; 9.20 × 10^−4^ in the community of Piquiazal; and 7.55 × 10^−5^ in Vila Dnit (Table S4). The values exceed the acceptability limit in the regions of Vila Central and Piquiazal, indicating a relevant carcinogenic risk [46]. The presence of traces of DDT in these communities reflects its intensive use over decades, especially in Brazil, where it was widely used to control malaria-carrying pests. The proximity of Vila Central to indigenous lands and the presence of illegal gold mines increase local vulnerability to the spread of malaria, in a context of unsanitary conditions and poor health infrastructure, increasing exposure to chemicals. The indiscriminate use of DDT in the past, without adequate environmental and public health assessments, still has consequences today [75].

Regarding the evaluation of the insecticide alfacipermethrin, the community of Vila Dnit, in Porto Velho, had the highest ILCR rates, reaching values of up to 4.02 × 10^−7^ for soil ingestion in adolescents. In Mazagão, in the community of Vila Maracá, the maximum value was 3.08 × 10^−7^, while in Cantá, in the Vila Central region, it was 2.64 × 10^−7^. For the dietary route, the maximum values observed were 1.17 × 10^−1^ in children in Vila Dnit, 6.86 × 10^−2^ in Vila Maracá, and 5.05 × 10^−2^ in Vila Central. All these values exceed the acceptability limit of 1 × 10^−4^, indicating a potential carcinogenic risk associated with exposure to alfacipermethrin in all regions studied [46].

Although the results suggest that alfacipermethrin, widely used in LLINs and indoor spraying, may pose carcinogenic risks, there are no conclusive studies confirming specific carcinogenic effects of this insecticide. However, evidence indicates that maternal exposure to pyrethroids may compromise the cognitive development and motor skills of children, especially girls [76].

Despite the effectiveness of alfacipermethrin-impregnated LLINs in controlling malaria vectors, inappropriate use of these physical barriers can result in unsafe exposure. Previous studies have shown that children are particularly vulnerable to oral exposure to insecticides impregnated in mosquito nets, which reinforces the need for monitoring and control of the use of these interventions [22,77].

With regard to non-carcinogenic risks, the community of Vila Central stood out for having the highest total HQ and HI values, both for soil and food intake. Among the contaminants evaluated, the highest HQ was observed for the *p,p′*-DDD analyte in the age groups of adolescents (4.83 × 10^−4^), children (4.82 × 10^−4^), and adults (2.96 × 10^−4^), in descending order (Table S5). These values indicate that there is no risk to human health, since all remain below the threshold of concern of 1. On the other hand, in the assessment of food intake, the *p,p′*-DDT analyte presented significantly higher HQ values, reaching up to 17.30 in children, 10.90 in adolescents, and 6.73 in adults. These results suggest the possibility of health risks from non-carcinogenic effects, since these values exceeded the threshold of concern (HQ > 1).

Regarding the total HI results presented in Table S5, soil intake revealed the highest values in children and adolescents, both with 1.49 × 10^−3^, followed by adults with 9.13 × 10^−4^. All these values are below the risk threshold (HI < 1), indicating no health risk in this context. However, in the assessment of food intake, the highest HI was observed in children (336.61), followed by adolescents (212.5) and adults (131.58). These values exceed the extreme risk threshold (HI > 4), indicating significant concern about continued exposure to the contaminants analyzed and the potential impact on health. Similar values were observed in municipalities such as Mazagão and Porto Velho, which also exceeded this limit, reinforcing the need for attention to the possible health consequences of this exposure.

Exposure to DDTs can cause damage to the nervous, immune, and reproductive systems, with women being particularly vulnerable due to their greater amount of adipose tissue and sensitivity to endocrine disruptors [78]. In addition, exposure to alphacypermethrin has the potential to cause adverse effects on male reproductive health, including hypospadias and testicular dysgenesis [79]. These findings underscore the importance of monitoring and mitigating exposure to these compounds to protect public health.

#### 3.6.2. Contribution of Individual Exposure Routes

In the assessment of the main routes of exposure to carcinogenic risk, soil ingestion presented ILCRs ranging from 5.98 × 10^−9^ to 4.02 × 10^−7^, values considered within the acceptable range, as they are below the limit of 1 × 10^−4^. On the other hand, food intake presented ILCRs ranging from 3.49 × 10^−4^ to 1.7 × 10^−1^, indicating a potential cancer risk for the population, since the values exceeded the limit of concern (ILCR > 1 × 10^−4^) [46].

Regarding non-carcinogenic risk, HQ values for soil ingestion ranged from 3.91 × 10^−6^ to 4.43 × 10^−4^, all below the threshold of concern of 1, indicating no risk to human health through this route. For food intake, HQ values ranged from 3.45 × 10^−1^ to 312, suggesting a potential health risk from non-carcinogenic effects associated with the intake of contaminated food, since some values exceeded the limit of 1 [80].

In general, food intake has been identified as the main route of exposure to both carcinogenic and non-carcinogenic health risks, with children being the most vulnerable. Consumption of breast milk, which can carry lipophilic contaminants such as DDT, is an important factor in this vulnerability, since DDT bioaccumulates in adipose tissue and can be transmitted to children during breastfeeding [81]. A study reports that maternal exposure to DDT, especially during pregnancy and breastfeeding, may increase the risk of obesity in future generations [82].

#### 3.6.3. Individual Contribution of DDTs to Health Risks

Regarding the individual contribution of DDT metabolites, *p,p′*-DDT was the analyte with the highest contribution to carcinogenic risk, followed by *o,p′*-DDT, *o,p′*-DDD, *p,p′*-DDD, *o,p′*-DDE, and *p,p′*-DDE. For non-carcinogenic risk, *p,p′*-DDT was also the main contributor, followed, in decreasing order, by the analytes *o,p′*-DDD, *p,p′*-DDD, *o,p′*-DDT, *o,p′*-DDE, and *p,p′*-DDE. In an investigation carried out in China, the op′ DDT isomer stood out for carcinogenic risk and *p,p′*-DDD for non-carcinogenic health risk. It is important to emphasize that the degradation of DDT in the environment depends on several local environmental conditions [10].

Among the DDT metabolites, *o,p′*-DDT and *p,p′*-DDE stand out for their health risks. *o,p′*-DDT can increase uterine weight in women and antagonize androgen receptors in men, while *p,p′*-DDE inhibits the binding of testosterone to these receptors, interfering with endocrine processes [83]. DDT, due to its persistence in the environment and in the fat of organisms, continues to pose a significant risk to exposed communities. Exposure to DDT may increase the risk of early breast cancer, especially due to its estrogenic properties, which are associated with breast cancer [84].

Given its continued impact, rigorous environmental monitoring and effective regulatory actions are essential to protect the health of exposed populations. Furthermore, it is crucial to promote education on the safe use of pesticides and the routes of exposure to these substances, ensuring human health and environmental preservation [85,86].

## 4. Conclusions

The findings of this study show the persistence of pesticides in Amazonian soil and point to the presence of contemporary sources of contamination. The results of exposure to health risks reinforce the importance of considering cumulative exposure and the specific vulnerabilities of the local population, particularly sensitive groups such as children.

Beyond the specific characterization of contamination, the data obtained indicate the need for integrated approaches to environmental monitoring and risk assessment, with an emphasis on multiple routes of exposure. Future investigations should prioritize the development of spatial and temporal models of contaminant dispersion, the monitoring of complementary environmental matrices, such as water, as well as ecotoxicological studies applied to bioindicator species in the region. These initiatives are essential to support effective public policies for environmental control and prevention of health impacts in contexts of prolonged exposure to persistent pesticides.

## Figures and Tables

**Table 1:** Mean, standard deviation, maximum, and minimum values of DDT and metabolites in mg/kg.

Municipalities	*p,p′*-DDT X ^a^ ± S ^b^ (min–max) ^c^	*o,p′*-DDT X ± S (min–max)	*p,p′*-DDE X ± S (min–max)	*o,p′*-DDE X ± S (min–max)	*p,p′*-DDD X ± S (min–max)	*o,p′*-DDD X ± S (min–max)	∑DDT X ± S (min–max)
Mazagão	0.079 ± 0.087 (<LD ^d^ − 0.245)	0.045 ± 0.077 (<LD − 0.320)	0.002 ± 0.003 (<LD − 0.013)	<LD	0.005 ± 0.006 (<LD − 0.022)	0.003 ± 0.002 (<LD − 0.007)	2.219 0.139 ± 0.159 (<LD − 0.598)
Porto Velho	0.031 ± 0.061 (<LD − 0.193)	0.008 ± 0.012 (<LD − 0.030)	0.001 ± 0.002 (<LD − 0.007)	<LD	0.003 ± 0.003 (<LD − 0.009)	0.004 ± 0.005 (<LD − 0.019)	0.510 0.051 ± 0.072 (<LD − 0.230)
Cantá	0.128 ± 0.354 (<LD − 1.297)	0.055 ± 0.125 (<LD − 0.452)	0.002 ± 0.007 (<LD − 0.024)	<LD	0.013 ± 0.032 (<LD − 0.119)	0.004 ± 0.006 (<LD − 0.023)	2.694 0.207 ± 0.519 (<LD − 1.919)
Conama No. 420/2009	2 mg/kg		1 mg/kg		3 mg/kg		-

Caption: ^a^ mean; ^b^ standard deviation; ^c^ minimum and maximum values; ^d^ below the detection limit.

**Table 2:** Pearson correlation of DDT, DDD, DDE, pH, and OM.

	*p,p′*-DDT	*o,p′*-DDT	*p,p′*-DDE	*p,p′*-DDD	*o,p′*-DDD	pH
*o,p′*-DDT	0.864					
*p,p′*-DDE	0.866	0.947				
*p,p′*-DDD	0.965	0.894	0.910			
*o,p′*-DDD	0.683	0.672	0.694	0.728		
pH	-*	-	-	-	-	
OM	-	-	-	-	-	0.416

-*: Significance criterion not acceptable.

## Data Availability

The original contributions presented in this study are included in the article. Further inquiries can be directed to the corresponding author.

## Supplementary Materials

The following supporting information can be downloaded at https://www.mdpi.com/article/10.3390/toxics13100900/s1, Equation (S1): Calculation of the concentration in % of organic matter (OM) in the soil; Equation (S2): Average daily dose (ADD, mg/kg/day) via soil ingestion (ADDInt_soil); Equation (S3): Average daily dose (ADD, mg/kg/day) via food ingestion (ADDInt_food); Equation (S4): Calculation of incremental lifetime cancer risk (ILCR); Equation (S5): Calculation of the non-carcinogenic risk ratio (HQ) for the soil ingestion (HQInt_soil) and food ingestion (HQInt_food) exposure pathways; Equation (S6): Calculation of the total risk index (HI) for non-cancer risk; Figure S1: Average daily dose (ADD. mg/kg/day) of DDTs and alphacipermethrin transmitted by the routes of exposure: (**A**) soil ingestion and (**B**) food ingestion; Figure S2: Results of granulometric analysis of soil granulometry in the Piquiazal and Vila Maracá communities; Figure S3: Results of granulometric analysis of soil granulometry in the Vila Dnit community; Figure S4: Results of granulometric analysis of soil granulometry of the Serra Grande and Vila Central communities; Figure S5: Principal component analysis (PCA) of the variables evaluated in this study; Table S1: Soil quality guidelines for DDT (total) (mg/kg) [41]; Table S2: Bioconcentration factors of sum DDTs for different vegetables in different sites [45]; Table S3: Results of the concentrations (mg/kg) of the pesticides and measurement of the pH and OM of the soil; Table S4: Results for incremental lifetime cancer risk (ILCR) for intake from soil and food, for child, teen, and adult; Table S5: Results for the hazard quotient (HQ) for non-carcinogenic risk and for the hazard index (HI) to estimate the total non-carcinogenic risk for intake from soil and food, for child, teen, and adult.
