# Supplementary material for: Potential Risks to Human Health Caused by the Use of Pesticides in Soils of Three Municipalities Impacted by Localized Malaria in the Brazilian Amazon
Source: Toxics. 2025 Oct 21;13(10):900. doi: 10.3390/toxics13100900 (PMC12567565; doi:10.3390/toxics13100900)
Supplement: Supplementary file 1 [file toxics-13-00900-s001.zip › toxics-3771286-supplementary.pdf]

Supplementary Materials

Equation 1. Calculation of the concentration in % of Organic Matter (OM) in the soil.

$$OM(\%) = \left(\frac{m(g) greenhouse\ dry\ soil - m(g) soil\ burned\ in\ a\ muffle\ furnace}{m(g) greenhouse\ dry\ soil}\right) x\ 100$$

m: mass
OM: Organic Matter

Equation 2. Average daily dose (ADD, mg/kg/day) via the soil ingestion (ADDInt_soil).

$$ADD_{Int\_soil} = \frac{C_{soil}\ x\ IR_{soil}\ x\ ED\ x\ EF\ x\ CF}{BW\ x\ AT}$$

ADD_{Int_soil}: average daily dose - soil ingestion (mg/kg/day)
$C_{soil:}$ concentration of DDTs and Alphacypermethrin in the soil ($mg.kg^{-1}$)
$IR_{soil:}$ soil ingestion rate (mg/d)
ED: duration of exposure (year)
EF: exposure frequency (d/year)
CF: conversion factor (kg/mg)
BW: body weight of exposed individual (kg)
AT: average life expectancy (d)

Equation 3. Average daily dose (ADD, mg/kg/day) via the food ingestion (ADDInt_food).

$$ADD_{Int\_food} = \frac{C_{soil}\ x\ BCF\ x\ IR_{vegetable}\ x\ ED\ x\ EF}{BW\ x\ AT}$$

ADD_{Int_food}: average daily dose - food ingestion (mg/kg/day)
$C_{soil:}$ concentration of DDTs and Alphacypermethrin in the soil ($mg.kg^{-1}$)
BCF: bioconcentration factor (no unit)
$IR_{vegetable}$: vegetable intake rate (kg/d)
ED: duration of exposure (year)
EF: exposure frequency (d/year)
BW: body weight of exposed individual (kg)
AT: average life expectancy (d)

Equation 4. Calculation of Incremental Lifetime Cancer Risk (ILCR).

$$ILCR = ADD\ x\ SF$$
$$ILCRs = \sum ILCR$$

ILCR: incremental lifetime cancer risk
ADD: average daily dose (mg/kg/day)
SF: oral slope of the factor for ingestion $(mg/kg/d)^{-1}$

Equation S5. Calculation of the non-carcinogenic risk ratio (HQ) for the soil ingestion (HQInt_soil) and food ingestion (HQInt_food) exposure pathways.

$$HQ = \frac{ADD}{RfD}$$

HQ: rate of non-cancer risk to human health.
ADD: average daily intake (mg/kg/day).
RfD: reference dose for the specific chemical exposure route (mg/kg/day).

Equation S6. Calculation of the non-carcinogenic risk ratio (HQ) for the soil ingestion (HQInt_soil) and food ingestion (HQInt_food) exposure pathways.

$$HI = \sum HQ$$

HI: non-cancer risk index for human health.
HQ: rate of non-carcinogenic risk to human health.

Figure S1. Average daily dose (ADD. mg/kg/day) of DDTs and alphacipermethrin transmitted by the routes of exposure: (A) soil ingestion and (B) food ingestion.

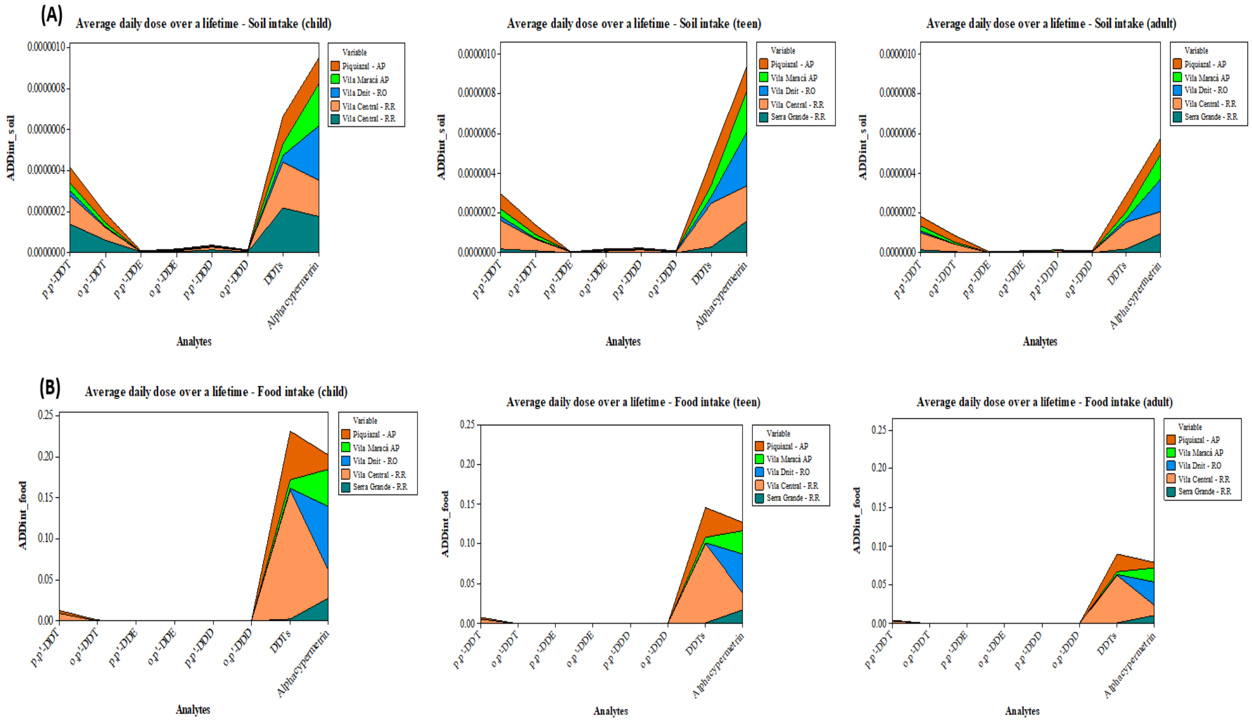

Figure S2. Results of granulometric analysis Soil granulometry in the Piquiazal and Vila Maracá communities.

.

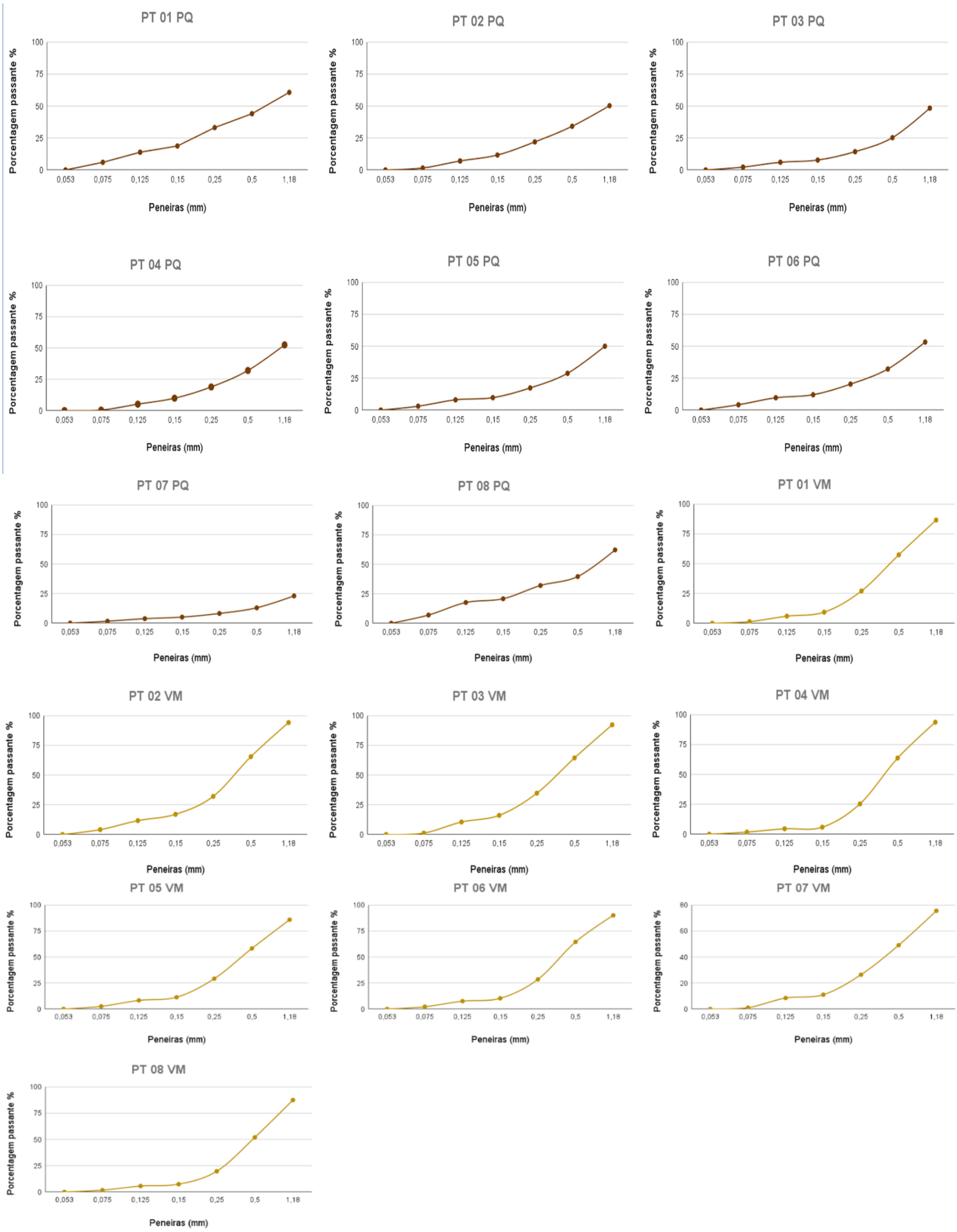

Figure S3. Results of granulometric analysis Soil granulometry in the Vila Dnit community.

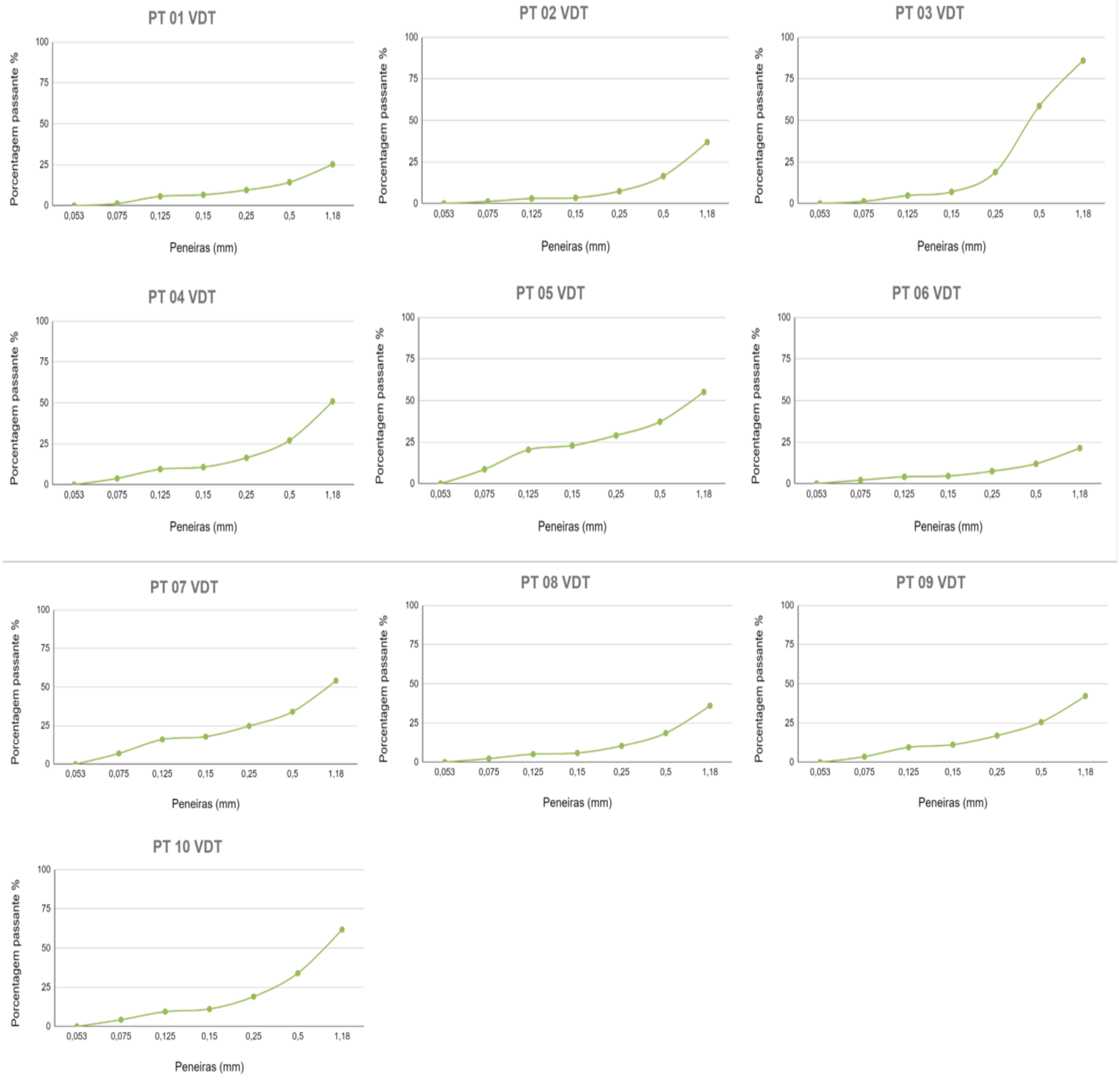

Figure S4. Results of granulometric analysis Soil granulometry of the Serra Grande and Vila Central communities.

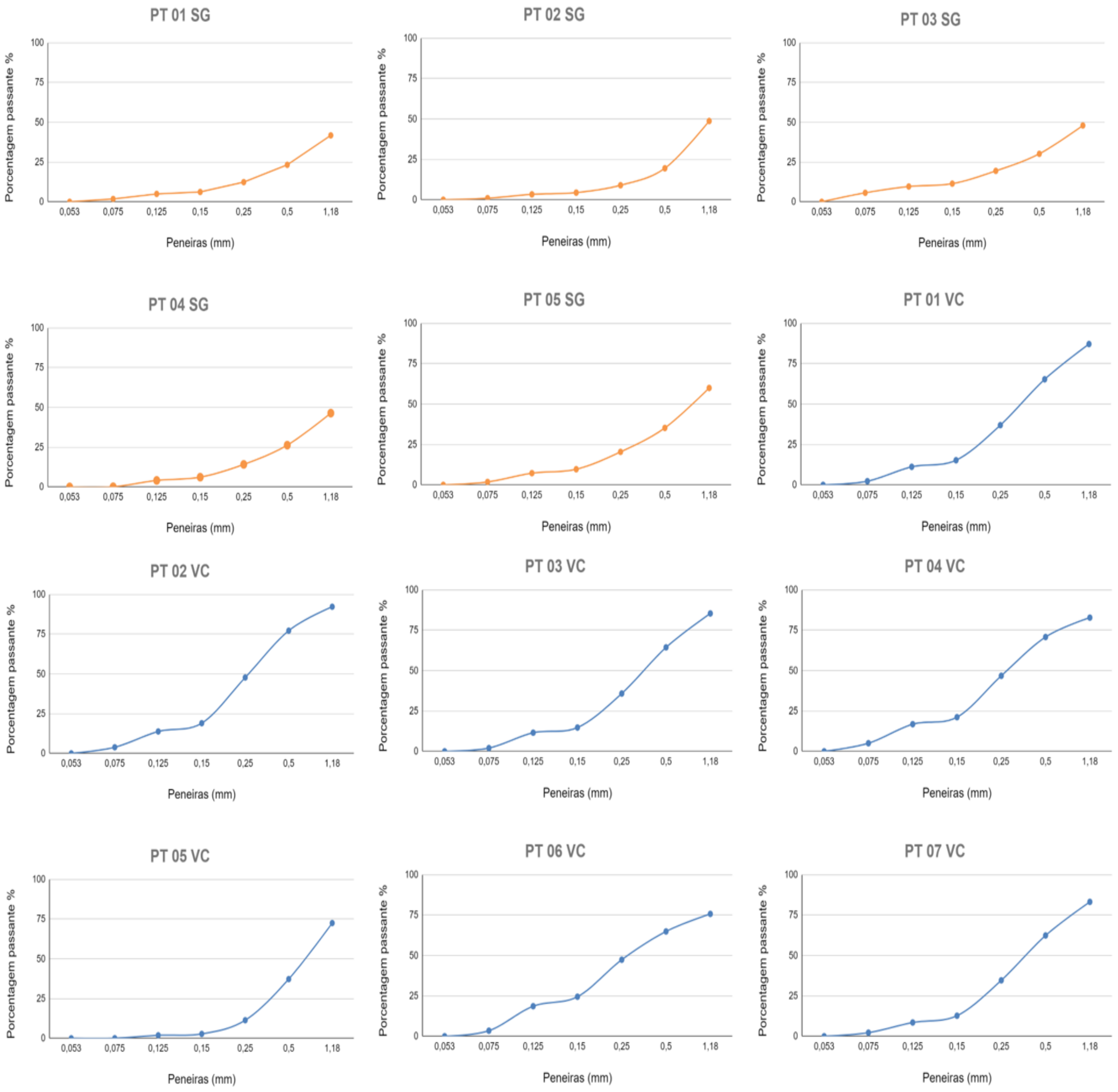

Figure S5. Principal component analysis (PCA) of the variables evaluated in the study.

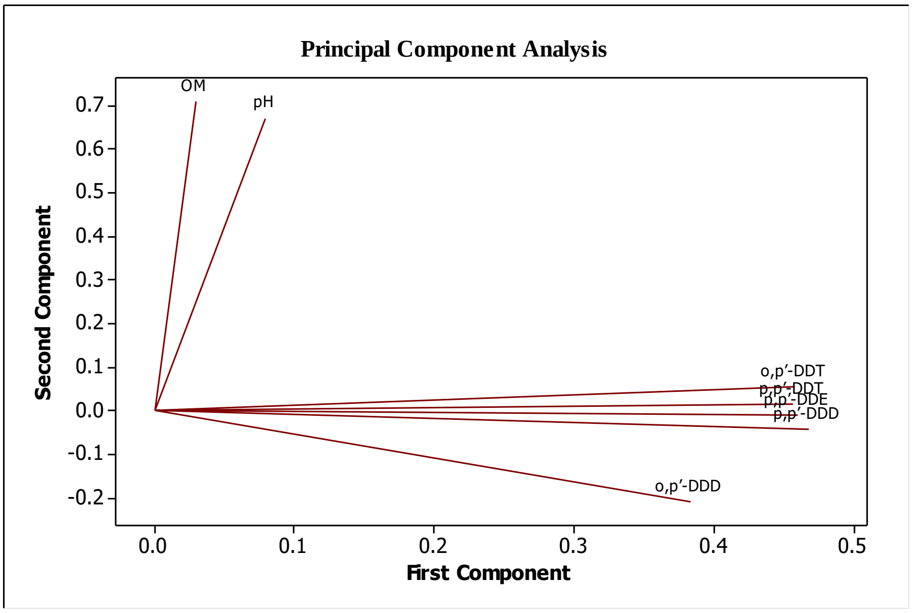

Table S1. Soil quality guidelines for DDT (total) (mg/kg) [41].

	Land use			
	**Agricultural**	**Residential/ parkland**	**Commercial**	**Industrial**
**Guideline**	**0.7[a]**	**0.7[a]**	**12[a.b]**	**12[a.b]**
SQGHH	NC[c]	NC[c]	NC[c]	NC[c]
Limiting pathway for SQGHH	ND	ND	ND	ND
Provisional SQGHH	NC[d]	NC[d]	NC[d]	NC[d]
Limiting pathway for provisional SQGHH	ND	ND	ND	ND
SQGE	0.7[e]	0.7[e]	12[e]	12[e]
Limiting pathway for SQGE	Soil and food ingestion	Soil and food ingestion	Soil contact	Soil contact
Provisional SQGE	NC[f]	NC[f]	NC[f]	NC[f]
Limiting pathway for provisional SQGE	ND	ND	ND	ND
Interim soil quality criterion (CCME 1991)	-	-	-	-

Notes: NC = not calculated; ND = not determined; SQGE = soil quality guideline for environmental health; SQGHH = soil quality guideline for human health.

[a]Data are sufficient and adequate to calculate only an SQGE. An interim soil quality criterion (CCME 1991) has not been established for this land use. therefore. the SQGE becomes the soil quality guideline.

[b]In site-specific situations where the size and/or the location of commercial and industrial land uses may impact primary. secondary or tertiary consumers. the soil and food ingestion guideline is recommended as the SQGE.

[c]There is no SQGHH for this land use at this time.
[d]There is no provisional SQGHH for this land use at this time.
[e]The environmental groundwater check (aquatic life) value has not been applied in the determination of the soil quality guideline. The applicability of the groundwater check (aquatic life) should be determined on a site-specific basis.
[f]Because data are sufficient and adequate to calculate an SQGE for this land use. a provisional SQGE is not calculated.

Table S2. Bioconcentration factors of sum DDTs for different vegetables in different sites location [45].

Collection Site	Vegetables	Vegetables BCF (DDTs)
BHG	sweet potato	0.00
	eggplant	0.46
	tomato	0.29
	radish	0.96
	sweet pepper	0.08
	cucumber	0.00
	red pepper	1.92
Niaga	turnip	0.32
	eggplant	0.68
	cabbage	0.67
	onion	0.61
	tomato	0.01
SHG	lettuce	0.00
	Sweet pepper	0.00
	tomato	0.00
Mbao	sweet pepper	0.00
	cabbage	0.00
	tomato	0.00
	turnip	0.07
Malika	Cabbage	0.00
	sweet pepper	0.00
GNP	tomato	0.00

.

Table S3. Results of the concentrations (mg/kg) of the pesticides and measurement of the pH and OM of the soil.

Municipalities	Sites	*p,p′*-DDT	*o,p′*-DDT	*p,p′*-DDE	*o,p′*-DDE	*p,p′*-DDD	*o,p′*-DDD	∑DDT	alphacypermethrin	pH	OM (%)
Mazagão	PT 01 PQ	0.2314	0.3205	0.0129	<LD	0.0220	<LQ	0.5984	0.2521	5.46	10.76
	PT 02 PQ	0.0505	0.0170	0.0022	<LD	0.0047	<LD	0.0813	0.2426	5.48	13.08
	PT 03 PQ	0.0161	<LD	<LD	<LD	<LD	<LD	0.0243	0.1105	6.69	12.41
	PT 04 PQ	0.0801	0.0452	<LD	<LD	0.0065	<LD	0.1388	0.1262	4.72	19.74
	PT 05 PQ	0.2170	0.0623	0.0025	<LD	0.0093	<LD	0.2980	0.1892	6.18	15.86
	PT 06 PQ	0.2446	0.0637	0.0016	<LD	0.0153	<LD	0.3322	0.1675	6.12	15.21
	PT 07 PQ	0.0028	0.0127	<LD	<LD	0.0015	<LD	0.0240	0.1086	7.34	11.36
	PT 08 PQ	0.0159	0.0150	0.0026	<LD	0.0012	<LD	0.0417	0.1909	6.44	14.30
	PT 01 VM	0.1055	0.0461	0.0005	<LD	0.0035	<LD	0.1625	0.3108	7.35	5.31
	PT 02 VM	0.0640	0.0298	<LD	<LD	0.0035	<LQ	0.1090	0.2929	5.79	3.67
	PT 03 VM	<LD	<LD	<LD	<LD	0.0013	<LD	0.0095	0.7748	5.42	4.45
	PT 04 VM	0.0014	<LD	0.0014	<LD	0.0015	<LD	0.0122	0.1526	6.74	2.81
	PT 05 VM	0.0692	0.0310	<LD	<LD	0.0056	<LD	0.1129	0.1527	6.77	4.51
	PT 06 VM	0.1465	0.0602	0.0009	<LD	0.0050	<LQ	0.2243	0.2419	6.46	6.07
	PT 07 VM	0.0141	0.0161	<LD	<LD	0.0035	<LD	0.0409	0.1888	5.72	7.26
	PT 08 VM	<LD	<LD	<LD	<LD	0.0010	<LD	0.0092	0.1179	6.22	5.35
Porto Velho	VDT 01	0.0373	0.0240	0.0066	<LD	0.0034	<LD	0.0782	0.4397	3.91	6.44
	VDT 02	<LD	<LD	<LD	<LD	0.0011	<LD	0.0093	0.2523	5.03	2.00
	VDT 03	<LD	<LD	<LD	<LD	0.0010	<LD	0.0092	0.4357	4.35	0.58
	VDT 04	0.0648	0.0297	0.0004	<LD	0.0057	<LD	0.1076	0.4042	3.26	2.24
	VDT 05	0.0076	<LD	0.0009	<LD	<LD	0.0191	0.0337	0.4734	3.98	1.99
	VDT 06	0.1933	0.0195	0.0015	<LD	0.0091	<LD	0.2304	0.3284	3.39	2.22
	VDT 07	<LD	<LD	<LD	<LD	0.0010	<LD	0.0092	0.3375	4.11	2.70
	VDT 08	<LD	<LD	<LD	<LD	0.0012	<LD	0.0094	0.3403	3.40	2.44
	VDT 09	<LD	<LD	<LD	<LD	0.0009	<LD	0.0091	0.2944	3.38	2.20
	VDT 10	0.0040	<LD	<LD	<LD	0.0014	<LD	0.0136	0.3382	4.26	2.05
Cantá	PT 01 VC	1.2972	0.4520	0.0236	<LD	0.1190	0.0225	1.9194	0.1844	5.93	3.31

	PT 02 VC	0.0684	0.0120	<LD	<LD	0.0058	<LD	0.0933	0.2417	5.85	2.27
	PT 03 VC	0.1231	0.0488	<LD	<LD	0.0021	<LD	0.1812	0.3409	5.29	5.22
	PT 04 VC	0.0246	0.1326	0.0056	<LD	0.0251	0.0086	0.2015	0.2853	4.95	4.08
	PT 05 VC	0.0039	<LD	<LD	<LD	<LD	<LD	0.0120	0.1205	5.37	3.90
	PT 06 VC	<LD	<LD	<LD	<LD	0.0015	<LD	0.0097	0.1618	4.40	3.72
	PT 07 VC	<LD	<LD	<LD	<LD	0.0016	<LD	0.0098	0.2415	4.20	4.44
	PT 08 VC	0.0156	0.0094	<LD	<LD	0.0022	<LD	0.0343	0.3388	5.24	3.75
	PT 01 SG	<LD	<LD	<LD	<LD	0.0013	<LD	0.0095	0.1001	5.22	4.42
	PT 02 SG	0.0422	0.0153	<LD	<LD	0.0025	<LD	0.0672	0.1856	5.48	2.37
	PT 03 SG	<LD	0.0062	<LD	<LD	0.0013	<LD	0.0147	0.2032	4.14	3.95
	PT 04 SG	<LD	<LD	<LD	<LD	0.0013	<LD	0.0095	0.2839	4.56	4.33
	PT 05 SG	0.0920	0.0272	<LD	<LD	0.0058	<LD	0.1321	0.3077	5.05	3.44
	PT 01 VC	1.2972	0.4520	0.0236	<LD	0.1190	0.0225	1.9194	0.1844	5.93	3.31
**LD**		0.0001	0.0010	0.0001	0.0050	0.0001	0.0020	-	0.0003	-	-
**LQ**		0.0003	0.0033	0.0003	0.0165	0.0003	0.0066	-	0.0010	-	-
**Conama No 420/2009**		2 $mg.kg^{-1}$		1 $mg.kg^{-1}$		3 $mg.kg^{-1}$		-	-	-	-

Table S4. Results for Incremental lifetime cancer risk (ILCR) for intake soil and food, for child, teen and adult.

ILCR	Municipalities	Communities	Age group	Analytes							
				p,p′- DDT	*o,p′*-DDT	*p,p′*-DDE	*o,p′*-DDE	*p,p′*-DDD	*o,p′*-DDD	DDTs	Alphacypermethrin
ILCR soil Ingestion	Mazagão	Piquiazal	Child	2.68E-08	1.67E-08	6.89E-10	1.25E-09	1.33E-09	4.45E-10	4.63E-08	1.91E-07
			Teen	2.68E-08	1.68E-08	6.91E-10	1.25E-09	1.34E-09	4.46E-10	4.64E-08	1.91E-07
			Adult	1.64E-08	1.03E-08	4.23E-10	7.66E-10	8.19E-10	2.73E-10	2.84E-08	1.17E-07
		Vila Maracá	Child	1.25E-08	5.72E-09	8.98E-11	1.25E-09	5.49E-10	4.38E-10	1.93E-08	3.07E-07
			Teen	1.25E-08	5.74E-09	9.01E-11	1.25E-09	5.50E-10	4.39E-10	1.94E-08	3.08E-07
			Adult	7.68E-09	3.52E-09	5.52E-11	7.66E-10	3.37E-10	2.69E-10	1.19E-08	1.89E-07
	Porto Velho	Vila Dnit	Child	7.67E-09	1.84E-09	2.41E-10	1.25E-09	4.42E-10	6.53E-10	1.08E-08	4.01E-07
			Teen	7.69E-09	1.85E-09	2.42E-10	1.25E-09	4.43E-10	6.55E-10	1.08E-08	4.02E-07
			Adult	4.71E-09	1.13E-09	1.48E-10	7.66E-10	2.71E-10	4.01E-10	6.65E-09	2.46E-07
	Cantá	Vila Central	Child	4.78E-08	2.04E-08	9.29E-10	1.25E-09	3.47E-09	9.50E-10	7.50E-08	2.63E-07
			Teen	4.79E-08	2.05E-08	9.32E-10	1.25E-09	3.48E-09	9.53E-10	7.52E-08	2.64E-07
			Adult	2.94E-08	1.26E-08	5.71E-10	7.66E-10	2.13E-09	5.84E-10	4.61E-08	1.62E-07
		Serra Grande	Child	6.71E-09	2.44E-09	2.49E-11	1.25E-09	4.30E-10	3.52E-10	9.74E-09	2.38E-07
			Teen	6.73E-09	2.45E-09	2.50E-11	1.25E-09	4.31E-10	3.53E-10	9.76E-09	2.39E-07
			Adult	4.12E-09	1.50E-09	1.53E-11	7.66E-10	2.64E-10	2.16E-10	5.98E-09	1.46E-07
ILCR food ingestion	Mazagão	Piquiazal	Child	9.20E-04	1.06E-04	1.27E-07	3.57E-07	1.61E-06	6.60E-06	2.02E-02	2.65E-02
			Teen	5.80E-04	6.66E-05	8.01E-08	2.25E-07	1.02E-06	4.16E-06	1.27E-02	1.67E-02
			Adult	3.59E-04	4.12E-05	4.96E-08	1.39E-07	6.30E-07	2.57E-06	7.88E-03	1.03E-02
		Vila Maracá	Child	2.00E-04	1.24E-05	2.16E-09	3.57E-07	2.74E-07	2.72E-06	3.52E-03	6.86E-02
			Teen	1.26E-04	7.80E-06	1.36E-09	2.25E-07	1.73E-07	1.71E-06	2.22E-03	4.32E-02
			Adult	7.82E-05	4.83E-06	8.42E-10	1.39E-07	1.07E-07	1.06E-06	1.37E-03	2.68E-02
	Porto Velho	Vila Dnit	Child	7.55E-05	1.28E-06	1.55E-08	3.57E-07	1.77E-07	2.27E-06	1.10E-03	1.17E-01
			Teen	4.76E-05	8.08E-07	9.79E-09	2.25E-07	1.12E-07	1.43E-06	6.96E-04	7.37E-02
			Adult	2.95E-05	5.00E-07	6.06E-09	1.39E-07	6.92E-08	8.84E-07	4.31E-04	4.56E-02
	Cantá	Vila Central	Child	2.93E-03	1.58E-04	2.31E-07	3.57E-07	1.09E-05	2.29E-05	5.31E-02	5.05E-02
			Teen	1.85E-03	9.93E-05	1.46E-07	2.25E-07	6.89E-06	1.44E-05	3.35E-02	3.18E-02
			Adult	1.14E-03	6.15E-05	9.01E-08	1.39E-07	4.27E-06	8.92E-06	2.07E-02	1.97E-02
		Serra Grande	Child	5.78E-05	2.25E-06	1.66E-10	3.57E-07	1.68E-07	1.10E-06	8.95E-04	4.11E-02
			Teen	3.64E-05	1.42E-06	1.05E-10	2.25E-07	1.06E-07	6.94E-07	5.64E-04	2.59E-02
			Adult	2.25E-05	8.79E-07	6.49E-11	1.39E-07	6.55E-08	4.29E-07	3.49E-04	1.60E-02

Table S5. Results for the Hazard Quotient (HQ) for non-carcinogenic risk and for the Hazard Indices (HI) to estimate the total non-carcinogenic risk for intake soil and food, for child, teen and adult.

**non-carcinogenic risk**	**Municipalities**	**Communities**	**Age group**	**Analytes**								**HI total**
				p,p′- DDT	*o,p′*-DDT	*p,p′*-DDE	*o,p′*-DDE	*p,p′*-DDD	*o,p′*-DDD	DDTs	Alphacypermethrin	
HQ soil ingestion	Mazagão	Piquiazal	Child	1.57E-04	9.84E-05	6.76E-06	1.22E-05	1.85E-04	6.18E-05	2.72E-04	6.36E-06	8.00E-04
			Teen	1.58E-04	9.87E-05	6.78E-06	1.23E-05	1.86E-04	6.19E-05	2.73E-04	6.38E-06	8.03E-04
			Adult	9.67E-05	6.05E-05	4.15E-06	7.51E-06	1.14E-04	3.79E-05	1.67E-04	3.91E-06	4.92E-04
		Vila Maracá	Child	7.35E-05	3.37E-05	8.81E-07	1.22E-05	7.62E-05	6.08E-05	1.14E-04	1.02E-05	3.81E-04
			Teen	7.37E-05	3.38E-05	8.83E-07	1.23E-05	7.64E-05	6.10E-05	1.14E-04	1.03E-05	3.82E-04
			Adult	4.52E-05	2.07E-05	5.41E-07	7.51E-06	4.68E-05	3.74E-05	6.98E-05	6.29E-06	2.34E-04
	Porto Velho	Vila Dnit	Child	4.51E-05	1.08E-05	2.36E-06	1.22E-05	6.14E-05	9.07E-05	6.36E-05	1.34E-05	3.00E-04
			Teen	4.52E-05	1.09E-05	2.37E-06	1.23E-05	6.15E-05	9.09E-05	6.38E-05	1.34E-05	3.00E-04
			Adult	2.77E-05	6.66E-06	1.45E-06	7.51E-06	3.77E-05	5.57E-05	3.91E-05	8.21E-06	1.84E-04
	Cantá	Vila Central	Child	2.81E-04	1.20E-04	9.11E-06	1.22E-05	4.82E-04	1.32E-04	4.41E-04	8.78E-06	1.49E-03
			Teen	2.82E-04	1.21E-04	9.14E-06	1.23E-05	4.83E-04	1.32E-04	4.43E-04	8.81E-06	1.49E-03
			Adult	1.73E-04	7.38E-05	5.60E-06	7.51E-06	2.96E-04	8.11E-05	2.71E-04	5.40E-06	9.13E-04
		Serra Grande	Child	3.95E-05	1.44E-05	2.45E-07	1.22E-05	5.97E-05	4.89E-05	5.73E-05	7.93E-06	2.40E-04
			Teen	3.96E-05	1.44E-05	2.45E-07	1.23E-05	5.99E-05	4.91E-05	5.74E-05	7.95E-06	2.41E-04
			Adult	2.42E-05	8.83E-06	1.50E-07	7.51E-06	3.67E-05	3.01E-05	3.52E-05	4.87E-06	1.48E-04
HQ food ingestion	Mazagão	Piquiazal	Child	5.41E+00	6.22E-01	1.25E-03	3.50E-03	2.24E-01	9.17E-01	1.19E+02	8.84E-01	127.06
			Teen	3.41E+00	3.92E-01	7.85E-04	2.20E-03	1.41E-01	5.78E-01	7.48E+01	5.57E-01	79.88
			Adult	2.11E+00	2.42E-01	4.86E-04	1.36E-03	8.74E-02	3.58E-01	4.63E+01	3.45E-01	49.44
		Vila Maracá	Child	1.18E+00	7.28E-02	2.12E-05	3.50E-03	3.80E-02	3.77E-01	2.07E+01	2.29E+00	24.66
			Teen	7.43E-01	4.59E-02	1.33E-05	2.20E-03	2.40E-02	2.38E-01	1.31E+01	1.44E+00	15.59
			Adult	4.60E-01	2.84E-02	8.25E-06	1.36E-03	1.48E-02	1.47E-01	8.08E+00	8.92E-01	9.62
	Porto Velho	Vila Dnit	Child	4.44E-01	7.55E-03	1.52E-04	3.50E-03	2.46E-02	3.15E-01	6.50E+00	3.90E+00	11.19
			Teen	2.80E-01	4.76E-03	9.60E-05	2.20E-03	1.55E-02	1.98E-01	4.09E+00	2.46E+00	7.05
			Adult	1.73E-01	2.94E-03	5.94E-05	1.36E-03	9.61E-03	1.23E-01	2.53E+00	1.52E+00	4.36
	Cantá	Vila Central	Child	1.73E+01	9.27E-01	2.26E-03	3.50E-03	1.52E+00	3.18E+00	3.12E+02	1.68E+00	336.61
			Teen	1.09E+01	5.84E-01	1.43E-03	2.20E-03	9.57E-01	2.00E+00	1.97E+02	1.06E+00	212.50
			Adult	6.73E+00	3.62E-01	8.83E-04	1.36E-03	5.93E-01	1.24E+00	1.22E+02	6.57E-01	131.58
		Serra Grande	Child	3.40E-01	1.33E-02	1.63E-06	3.50E-03	2.33E-02	1.53E-01	5.26E+00	1.37E+00	7.16
			Teen	2.14E-01	8.35E-03	1.03E-06	2.20E-03	1.47E-02	9.63E-02	3.32E+00	8.64E-01	4.52
			Adult	1.33E-01	5.17E-03	6.37E-07	1.36E-03	9.10E-03	5.96E-02	2.05E+00	5.35E-01	2.79
